# Educators’ behaviours during feedback in authentic clinical practice settings: an observational study and systematic analysis

**DOI:** 10.1186/s12909-019-1524-z

**Published:** 2019-05-02

**Authors:** Christina E. Johnson, Jennifer L. Keating, Melanie K. Farlie, Fiona Kent, Michelle Leech, Elizabeth K. Molloy

**Affiliations:** 1 Monash Doctors Education, Monash Health and Department of Medical Education, Melbourne Medical School, University of Melbourne, Melbourne, Victoria Australia; 2 Department of Physiotherapy, School of Primary and Allied Health Care, Faculty of Medicine Nursing and Health Science, Monash University, Melbourne, Australia; 3 Allied Health Workforce Innovation, Strategy, Education & Research (WISER) Unit, Monash Health, and School of Primary and Allied Health Care, Faculty of Medicine, Nursing and Health Sciences at Monash University, Melbourne, Australia; 4 Education Portfolio, Faculty Medicine, Nursing and Health Sciences, Monash University, Melbourne, Australia; 5 Monash School of Medicine, Faculty of Medicine, Nursing & Health Sciences, Monash University and Monash Health, Melbourne, Australia; 6 Department of Medical Education, Melbourne Medical School, University of Melbourne, Melbourne, Australia

**Keywords:** Feedback, Formative feedback, Effective feedback, Health professions education, Professional development

## Abstract

**Background:**

Verbal feedback plays a critical role in health professions education but it is not clear which components of effective feedback have been successfully translated from the literature into supervisory practice in the workplace, and which have not. The purpose of this study was to observe and systematically analyse educators’ behaviours during authentic feedback episodes in contemporary clinical practice.

**Methods:**

Educators and learners videoed themselves during formal feedback sessions in routine hospital training. Researchers compared educators’ practice to a published set of 25 educator behaviours recommended for quality feedback. Individual educator behaviours were rated 0 = not seen, 1 = done somewhat, 2 = consistently done. To characterise individual educator’s practice, their behaviour scores were summed. To describe how commonly each behaviour was observed across all the videos, mean scores were calculated.

**Results:**

Researchers analysed 36 videos involving 34 educators (26 medical, 4 nursing, 4 physiotherapy professionals) and 35 learners across different health professions, specialties, levels of experience and gender. There was considerable variation in both educators’ feedback practices, indicated by total scores for individual educators ranging from 5.7 to 34.2 (maximum possible 48), and how frequently specific feedback behaviours were seen across all the videos, indicated by mean scores for each behaviour ranging from 0.1 to 1.75 (maximum possible 2). Educators commonly provided performance analysis, described how the task should be performed, and were respectful and supportive. However a number of recommended feedback behaviours were rarely seen, such as clarifying the session purpose and expectations, promoting learner involvement, creating an action plan or arranging a subsequent review.

**Conclusions:**

These findings clarify contemporary feedback practice and inform the design of educational initiatives to help health professional educators and learners to better realise the potential of feedback.

## Background

Modern clinical training, aligned with competency based education and programmatic assessment, is focused on assessment and feedback on routine tasks in the workplace, targeting the highest level in Miller’s framework for competency assessment [1–3]. Feedback is one of the most powerful influences on learning and performance [4–8]. It offers the opportunity for a learner to benefit from another practitioner’s critique, reasoning, advice and support. Through this collaboration, the learner can enhance their understanding of what the performance targets are and how they can reach those standards [9, 10]. ‘On the run’ or informal feedback refers to brief fragments of feedback that occur in the midst of delivering patient care. A formal feedback session typically refers to a senior clinician (educator) and student or junior clinician (learner) discussing the learner’s performance in a more comprehensive fashion. Formal feedback sessions often occur as a mid- or end-of-attachment appraisal or as part of a workplace-based assessment. However the success of this model relies on everyday clinicians providing effective feedback. It is not clear which components of effective feedback have been successfully translated from the literature into supervisory practice in the workplace, and which have not. Information on gaps in translation could be used to better target professional development training, or to design strategies to overcome impediments to implementing quality feedback behaviours.

Studies involving direct observation of authentic feedback in hospitals are rare. Observational studies are highly valuable, as they provide primary evidence of what actually happens in everyday clinical education. Direct observation can be achieved either by researchers observing the activity or via video-observation. We identified only a few previous direct observation studies: these involved junior learners (medical students or junior residents) in a few specialties (internal or family medicine) involving formal or informal feedback (in outpatient clinics, on a ward, or following summative simulated clinical scenarios) [11–18]. An additional single study involved physiotherapy students during formal mid- or end-of-attachment feedback [19]. The scarcity of observational studies may be related to the time consuming nature, difficulty in arranging observers or video recording to coincide with feedback meetings slotted into busy schedules, or the reticence of participants to be observed or recorded. These studies reported that typically educators make comments on specific aspects of performance, teach important concepts, and describe or demonstrate how the learner can improve. However educators tend to speak most of the time, ask the learner for their self-assessment but then do not respond to it, avoid corrective comments and do not routinely create actions plans. However these findings may no longer reflect current practice. In addition, no study captured the diversity of clinical educators and learners that work in a hospital environment.

Therefore we set out to directly observe authentic formal feedback episodes in hospital training, via self-recorded videos, to review contemporary educators’ feedback practice in workplace-based learning environments. This could then clarify opportunities and inform the design of professional development training. In Australia, health professions training is concentrated in hospitals, integrating both inpatient wards and outpatient clinics; major dedicated specialist outpatient centres are rare and family medicine clinics are relatively small. We recruited a range of participants, characteristic of the diversity present in hospitals, as desirable feedback elements are not profession specific. We targeted formal feedback sessions to capture complete feedback interactions. We then analysed the composition of educators’ feedback practice using a comprehensive set of observable educator behaviours recommended for high quality feedback (see Table 1) [20]. This enabled a systematic analysis of the first set of data gathered using a comprehensive set of behavioural indicators, in contrast to previous studies in which less structured and more exploratory approaches were used. This framework outlines 25 discrete observable educator behaviours considered to enhance learner outcomes by engaging, motivating and assisting a learner to improve (see Table 1) [20]. This earlier publication by our team described how these items were developed, starting with an extensive literature review to identify distinct elements of an educator’s role substantiated by empirical information to enhance learner outcomes, then operationalised into observable behaviours and refined through a Delphi process with experts.

**Table 1:** Set of 25 educator behaviours that demonstrate high quality feedback in clinical practice

**Orientation and Process**	
1. *Based on observed performance*	
The educator’s comments were based on observed performance	
2. *Timely feedback*	
The educator offered to discuss the performance as soon as practicable	
3. *Feedback purpose clear*	
The educator explained that the purpose of feedback is to help the learner improve their performance	
4. *Establish a non-judgmental atmosphere: ‘here to help’*	
The educator indicated that while developing a skill, it is expected that some aspects can be improved and the educator is here to help, not criticise	
5. *Clarify feedback process, so learner knows what to expect*	
The educator described the intended process for the feedback discussion	
**Learner-centred Focus**	
6. *Encourage dialogue*	
The educator encouraged the learner to engage in interactive discussions	
7. *Seek learner’s priorities*	
The educator asked the learner about their learning priorities for the observation and feedback discussion, and responded to them	
8. *Encourage learner to ‘work it out for themselves’*	
The educator encouraged the learner to consider the issues and possible solutions during the feedback discussion	
9. *Encourage learner to focus on learning, rather than trying to cover up limitations*	
The educator encouraged the learner to discuss difficulties and ask questions regarding the performance so the educator could help the learner to develop solutions	
10. *Acknowledge learner’s emotional response*	
The educator acknowledged and responded appropriately to emotions expressed by the learner	
11. *‘Best interests at heart’*	
The educator showed respect and support for the learner	
**Performance Analysis**	
12. *Clarify the value of self-assessment*	
The educator asked what the learner understood about the benefits of self-assessment and helped clarify	
13. *Learner self-assessment*	
The educator asked the learner to identify key similarities and differences between the learner’s performance and the target performance	
14. *Target performance and reasoning clear*	
The educator clarified with the learner key features of the target performance and explained the reasoning	
15. *Educator assessment, including clear performance gap*	
The educator clarified with the learner similarities and differences between the learner’s performance and the target performance	
16. *Educator comments on a few, important issues*	
The educator’s comments focused on key issues for improving the performance	
17. *Specific instance (‘what happened’)*	
First the educator described, using neutral language, what the learner did (action, decision or behaviour), and the consequences	
18. *Educator’s perspective clear (‘why it matters’)*	
The educator clearly explained their perspective on the learner’s actions, including the reason for their concern	
19. *Educator explores learner’s perspective (‘why’ the learner acted as they did)*	
The educator explored the learner’s perspective and reasoning to reveal the basis for the learner’s actions (e.g. what was the learner trying to do and options considered/ difficulties encountered)	
20. *Focus on actions, not the person (‘did’ not ‘is’)*	
The educator’s comments were focused on the learner’s actions not personal characteristics	
**Action Plan**	
21. *Select learning priorities: most useful (important and relevant) for the learner*	
The educator helped the learner to select a couple of key aspects of the performance to improve	
22. *Develop the action plan: how to do it!*	
The educator helped the learner to work how they could improve their performance and specify the practical steps to achieve it	
23. *Check the learner understands the plans*	
The educator checked if the learner understood their learning goals and action plan, by asking them to summarise it in their own words	
24. *Checks the learner understands the rationale: ‘why it’s better’*	
The educator checked if the learner understood the rationale for their learning goals and action plan	
25. *Plan opportunities to review the impact of the feedback*	
The educator discussed with the learner possible subsequent opportunities for the learner to review their progress	

Reproduced with permission from Johnson *et al*. *BMC Medical Education* (2016) 16:96 Identifying educator behaviours for high quality verbal feedback in health professions education: literature and expert refinement

While we strongly endorse a learner-centred paradigm, we have chosen to focus on the educator’s role in feedback because educators are in a position of influence to create conditions that encourage learners to feel safe, participate and work out how to successfully improve their skills. We agree that specific feedback episodes are shaped by the individuals involved, the context and the culture, however strategies to promote a learner’s motivation and capability to enhance their performance remain relevant. Recommended feedback behaviours are not intended to be implemented in a robotic fashion but tailored to a particular situation by prioritising the most useful aspects throughout the interaction. The core segments of quality feedback include clarifying the target performance, analysing the learner’s performance in comparison to this target, outlining practical steps to improve and planning how to review progress [4, 9, 21]. Overarching themes include promoting motivation [22–25], active learning [26–28] and collaboration [29–32] within a safe learning environment [10, 33, 34].

### Research question

The research questions addressed in this study were:

1. What behaviours are exhibited by clinical educators in formal feedback sessions in hospital practice settings?
2. How closely do these behaviours align with published recommendations for feedback?

## Methods

### Research overview

In this observational study, senior clinicians (educators) observed junior clinicians or students (learners) performing routine clinical tasks in a hospital setting and then videoed themselves during the subsequent formal feedback session. We analysed each video using a check-list based on the set of educator behaviours recommended in high quality feedback (see Table 1) [20].

The feedback videos were captured at multiple hospitals within one of Australia’s largest metropolitan teaching hospital networks between August 2015 and December 2016. Ethics approval was obtained from the health service (Reference 15,233 L) and university human research ethics committees (Reference 2,015,001,338).

### Recruitment

Educators (senior clinicians) across medicine, nursing, physiotherapy, occupational therapy, speech therapy and social work, and their learners (either qualified health professionals undertaking further training or students), who were working with them, were invited to participate. A broad range of educators were sought, via widespread advertising of the study using flyers, emails circulated by unit administration assistants, short presentations at unit meetings and face-to-face meetings with staff across the health service. To be considered for participation, an educator had to contact the primary researcher (CJ), in response to the advertisement. Once an educator consented, they distributed flyers to any learners working with them, with instructions to contact the primary researcher (CJ) if the learners were interested in participating. Diversity was sought by rolling advertising to participants, with consideration of key factors including health profession and specialty, gender and supervisor experience (educators) or training level (learners). Once an educator and a learner had both consented, the pair were advised and they made arrangements to video a routine feedback session. They were asked to record an entire feedback encounter and aim for a duration of approximately 10 minutes but were not given any additional instructions regarding how to conduct the feedback session. Participants were not shown the set of 25 educator behaviours recommended for high quality feedback used to analyse the videos nor given any other education on feedback from the research team, as the aim was to study the nature of current feedback practices.

Consenting participants used a smart phone or computer to video-record themselves at their next scheduled formal feedback session related to either a workplace-based assessment or end-of-attachment performance appraisal. This video was subsequently uploaded to a password protected on-line drive and participants were instructed to delete their copy. The videos were numbered using a random number generator and the videos (other than the images) contained no personal identifying information.

### Video analysis

The group of raters were all health professionals (two medical, four physiotherapy) in senior education/educational research roles with extensive experience in supervision and feedback. Each rater analysed each video independently and compared their observations with the set of 25 educator behaviours recommended for high quality feedback (see Table 1) [20]. Each educator behaviour was rated 0 = not seen, 1 = done somewhat or done only sometimes, 2 = consistently done.

In a preparatory pilot study, we rated three videos using the instrument. We then met to discuss ratings and to identify differences in interpretation of items and the use of the rating scale. Strategies to encourage concordance and to clarify item meaning were developed. In particular we identified that Behaviour 2: *Timely feedback*: *The educator offered to discuss the performance as soon as practicable* was not observable, so it was excluded. For Behaviour 10: *Acknowledge learner’s emotional response: The educator acknowledged and responded appropriately to emotions expressed by the learner,* we decided that this would be rated as ‘2’ (consistently done) in the following situations i) if implicit or explicit indicators of learner emotion (such as anxiety or defensiveness) were detected, and the educator acknowledged, and attended to this, or ii) if emotional equilibrium was observed throughout the encounter, as we assumed that this emotional balance between educator and learner required the educator to be reading cues and acting accordingly. Subsequently the total item score could range from 0 to 48.

### Data analysis

The data provided two perspectives i) on an individual educator’s practice: how many of the behaviours recommended in high quality feedback were observed in each video and ii) across the whole group of educators: which behaviours were commonly performed. To characterise each individual educator’s practice seen in a video, the scores for each item were averaged across assessors and then summed to give a total score. To describe how commonly specific educator behaviours were observed amongst the whole group of educators, the mean score and standard devation for each item was calculated across all the videos [35]. To assess inter-rater reliability, total scores for each video were assessed for concordance between examiner pairs using Spearman’s rho.

## Results

Thirty-six feedback videos were available for analysis after five were excluded: two because they were incomplete (insufficient smartphone memory) and three because of technical errors with recording (audio unclear, time-lapse format used, participants not visible).

### Video participants

Thirty-four educators participated, with diversity across key characteristics (health profession and specialty, length of supervisor experience and gender). There were four nurses, four physiotherapists and 26 senior medical staff (three anaesthetists, three emergency physicians, two radiologists, one paediatrician, six physicians, three psychiatrists, three obstetrician-gynaecologists, one opthalmologist and four surgeons). There were 18 (52.9%) female and 16 (47.1%) male educators. Fourteen (41.2%) educators had 5 years or less educator experience, 11 (32.3%) had six to 10 years and 9 (26.5%) had more than 10 years.

Thirty-five learners participated with diversity across key characteristics (health profession and specialty, training level and gender). There were 9 (25.7%) students, 9 (25.7%) clinicians who were five years or less post-qualification, 15 (42.9%) clinicians 6 years or more post-qualification and 2 (5.7%) senior clinicians. Twenty-three learners were (65.7%) female and 12 (34.3%) were male. All participants were from the same health profession and specialty as their respective educators.

The feedback session was related to a mid- or end-of-attachment assessment in 11 (30.6%) videos and to a specific task (such as a procedural skill, clinical assessment, case discussion or presentation) in 25 (69.4%) videos. An official feedback form from an institution such as a university or specialist medical college was used in 11 (30.6%) of the feedback sessions, most of which were mid- or end-of-attachment assessments. Most of the assessments were formative but some were summative as a component of longitudinal training programs aligned with programatic assessment principles [3].

### Analysis of educator behaviours during feedback

Each video was analysed by four to six raters providing a total of 174 sets of ratings (unexpected time constraints on the project limited analysis by two raters). Missing data were uncommon (0.2% ratings missing).

#### Inter-rater reliability

To maximise data for comparison, the inter-rater reliability range for total scores was calculated for raters (4/6) who analysed all the videos: Spearman’s rho was 0.62–0.73. The other two raters rated 10 (28%) and 21 (58%) of the 36 videos and were not included in the inter-rater reliability analysis.

#### Individual educator’s feedback practice

To learn more about individual educator’s practice and how many of the recommended educator behaviours were observed in each video, we calculated a total score (sum of rating for each observed behaviour, averaged across all assessors) for each video. Total scores ranged from a minimum of 5.7 (11.9%) to a maximum of 34.2 (71.3%), with a mean score across educators of 22.5 (46.9%, SD 6.6), from a maximum possible score of 48. More detailed analysis (see Table 2) revealed virtually all the educators (88%) had a total score between 10 and 30. Although it was not our intention to compare performance across different characteristics (which would require sufficient sample sizes for each group, to enable comparisons), there seemed to be a fairly even spread of health professions, experience and gender across the score ranges.

**Table 2:** Range of total scores for individual educators (34 educators in 36 videos)

Total scores	Number of educators Total = 34	Health Profession M: medicine N: nursing P: physiotherapy	Supervisor experience (years) ≤ 5y ∣ 6–10 ∣ > 10y	Gender F: female M: male
40.1–48	0	0	0	0
30.1–40.0	3 (9%)	2 M 1 N	1 ∣ 1 ∣ 1	2F 1M
20.1–30.0	16^a^ (50%)	12 M 2 N 2P	8 ∣ 4 ∣ 4	7F 9M
10.1–20.0	14^a^ (38%)	11 M 1 N 2P	5 ∣ 6 ∣ 3	8F 6M
0–10.0	1 (3%)	1 M	0 ∣ 0 ∣ 1	1F

^a^included one educator who featured in two videos (mean total score used)

#### Frequency of specific educator behaviours across the whole group of educators

To explore how often specific feedback behaviours were observed amongst all participants, we calculated the mean rating score for each behaviour across all the videos. Table 3 displays the rating mean (SD) for each behaviour, ranked from most to least often observed. Some behaviours were seen in almost every video (highest mean rating 1.75, Behaviour 10) while others were very infrequently observed (lowest mean rating 0.05, Behaviour 25).

**Table 3:**
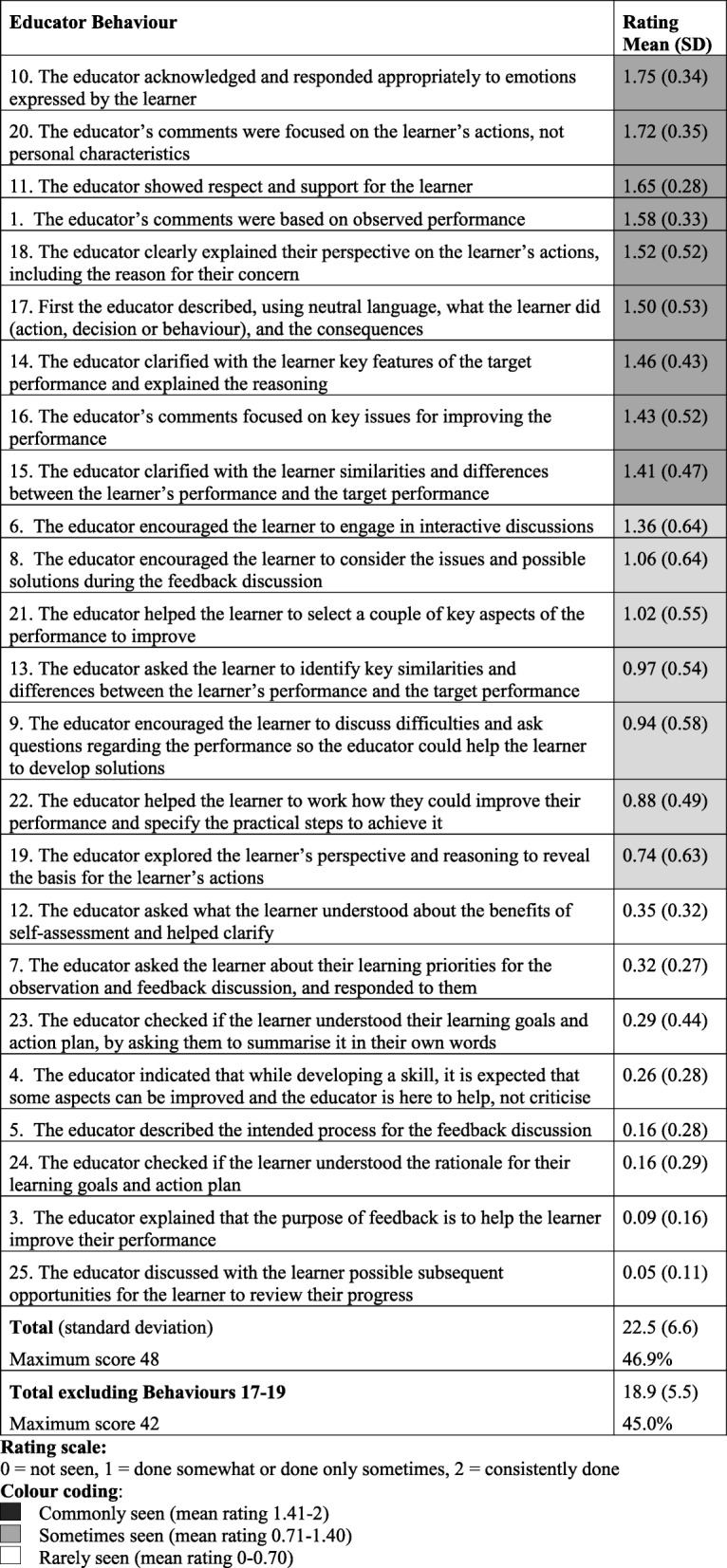
Observed educator behaviours ranked in order of rating, with the highest at the top. (after references)

Amongst those educator behaviours most commonly observed (top third: mean rating score 1.41–2.0), most related to the educator’s assessment of the learner’s performance. Educators commonly linked comments regarding learner performance to the learner’s actions (Behaviours 1, 17, 20), focused on important aspects for improvement (Behaviour 16), described similarities and differences between the learner’s performance and the target performance (Behaviour 15), and clarified what should be done and why (Behaviour 14). The other two behaviours commonly seen related to creating a safe learning environment. These included showing respect and support (Behaviour 11) and responding appropriately to emotions expressed by the learner (Behaviour 10).

The middle band of educator behaviours were seen intermittently (mean rating score 0.71–1.40) and related to educators encouraging learners to contribute their thoughts, opinions and ideas, and to reveal their uncertainties. These included encouraging the learner to participate in interactive discussions (Behaviour 6), try to work things out for themselves (Behaviour 8), analyse their own performance (Behaviour 13), reveal the reasoning behind their actions (Behaviour 19), raise difficulties and ask questions (Behaviour 9), and participate in choosing the most important aspects to improve (Behaviour 21) and practical ways to do this through an action plan (Behaviour 22).

The lowest band of educator behaviours were rarely seen (mean rating score 0–0.7) and primarily related to the set up and conclusion of a feedback session. At the start of the session, as part of creating a safe learning environment, the recommended educator behaviours included explicitly explaining that the purpose of the feedback was to help the learner improve (Behaviour 3), describing the proposed outline for the session (Behaviour 5), and stating their acceptance that mistakes are an inevitable part of the learning process (Behaviour 4). As part of the session conclusion or wrap-up, the recommended behaviours included checking a learner’s understanding of the learning goals and action plan (Behaviours 23, 24), and discussing future opportunities to review progress, to promote ongoing learning (Behaviour 25). The other educator behaviours that were rarely seen included the educator incorporating the learner’s learning priorities (Behaviour 7) and promoting the learner’s understanding of the value of their self-assessment (Behaviour 12).

## Discussion

In this study of educators’ feedback practice, we found considerable variation in both an individual educator’s practice and how frequently specific recommended behaviours were observed across the group of educators. This provides valuable insights into ‘what currently happens’ during formal feedback episodes in hospital-based training. These insights clarify opportunities for future research into educator development with the potential for substantial impact. Furthermore the recommended behaviours offer a repertoire of specific strategies that may assist educators to understand and enact these quality standards.

### Frequency of specific recommended behaviours observed across the group of educators

We found that educators routinely gave their assessment of the learner’s performance and described what the task should look like, but only intermittently asked learners for self-assessment or development of an action plan. This seems to reflect a culture in which the educator’s analysis of the learner’s performance predominates [36]. These findings echo those from earlier observational studies and feedback forms [11, 12, 17, 19, 37–40]. This suggests that typical feedback practice in the clinical setting has remained much the same since these omissions were last reported years ago.

Self-assessment is a key component in self-regulated learning and evaluative judgement, which promotes reflection, independent learning and achievement [28–30]. Invitations for learner self-asssessment provide learners with the opportunity to judge their work first and indicate what they most want help with [33, 41, 42]. Self-assessments can alert the educator to the potential for a negative emotional reaction and rejection of the educator’s opinion if the learner rates their performance much higher than the educator [43]. Self -assessment offer opportunities for learners to enhance their evaluative judgement by calibrating their understanding against an expert’s understanding of the observed performance and the desired performance standards [4, 44]. Recent work on student feedback literacy has highlighted the importance of strategically designing opportunities for learners to make judgements and discuss characteristics of quality work, to assist them to appreciate, interpret and utilise feedback [45].

The fact that an action plan continues to be frequently neglected similarly warrants serious attention. If educators do not support and guide learners to create an action plan, learners are left with the difficult task of working out by themselves how to transform feedback information into performance improvement [21]. Furthermore, when learners hear about performance gaps, their distress may be exacerbated if they do not know how to improve it [46].

Our study also identified a number of missing feedback features, which have not been previously documented. One involves positioning the development of a learner’s motivation, understanding and skills as the focal point for feedback. The literature suggests that a learner is only likely to successfully implement changes when they ‘wish to’ (motivation) and ‘know how to’ (clear understanding) [9, 29, 47, 48].

Self-determination theory argues that intrinsic motivation, which is associated with both higher performance and increased well-being, is promoted when a learner decides what to do, in line with their personal values and aspirations [23–25]. This is captured by recommended educator behaviours that position the learner as decision maker and the educator as guide (see Table 1: Behaviours 7, 21, 22). A learner must be convinced for themselves that the feedback is credible and valuable (Behaviours 1, 6, 7, 9, 20, 24) [8, 49, 50]. The free flow of information, opinion and ideas between the educator and learner creates a shared understanding, as a foundation for tailored advice and good decision making [51]. In addition, Goal Setting Theory asserts that a learner’s motivation is stimulated by a clear view of the performance gap, performance goals that are specific, achievable and valuable to the learner, and an action plan that is practical and tailored to suit their needs (Behaviours 14, 15, 21, 22) [22].

Recent advances in feedback have focused on the need to assist learners to process and utilise feedback information, so they ‘know how to’ enhance their performance. This is exemplified in the R2C2 feedback model, which includes assisting a learner to explore the information, their reactions to it and to design effective strategies for skill development [30, 32, 51]. Social constructivist learning theory describes how a learner makes meaning of new information through interactions with others [52]. To promote this active learning, recommended educator behaviours include encouraging the learner to analyse their own performance and ‘work things out for themselves’ (Behaviours 8,12,13), enquiring about the learner’s difficulties or questions (Behaviour 9) and checking the learner’s understanding of the action plan before concluding the session (Behaviours 23, 24) [53].

Another feature of effective feedback rarely seen in our study was educators deliberately setting up a safe learning environment at the start the session, although they showed respect and support for learners in general. Recent literature has reinforced the importance of promoting a safe learning environment and establishing an educational alliance [34]. This may be a particularly important strategy when the educator and learner do not have an established relationship, which seems to be increasingly commonplace in modern workplace training with short placements and multiple supervisors attending to learners [54]. Excessive anxiety negatively impacts on thinking, learning and memory [53, 55, 56]. Feedback is inherently psychologically risky; if a learner’s limitations are exposed, this can result in a lower grade or critical remarks from the educator, or threaten a learner’s sense of self [5, 33, 46]. Carless [10] highlighted the important role of trust in view of the strong relational, emotional and motivational influences of feedback. In an attempt to counter the natural anxiety, educators could be explicit that “mistakes are part of the skill-acquisition process” and that they desire to help, not to be critical [53]. In addition, if an educator negotiated the process and expectations for the feedback session, this could reduce the anxiety caused when the learner does not know, or have any control over, what is going to happen [30].

One final important feature was the isolation of the learning activity. In our study, no educator discused when or how the learner might be able to review to what extent they had been able to successfully develop the targeted skills (Behaviour 25); this was the lowest ranked behaviour of all. Molloy and Boud [9] have emphasised the importance of promoting performance development by linking learning activities, so that feedback plans can be implemented and progress evaluated in subsequent tasks. As supervision is increasingly short-term and fragmented in nature, collaborating with the learner in deliberately planning another opportunity to be assessed performing a similar task seems an important objective.

### Individual educator’s practice

The range in individual educator’s scores found in our study suggests the educators had variable expertise in feedback. Educators were not shown the check-list of recommended behaviours used in video analysis. Although not formally tested, there was no indication in the data that more experience conferred greater expertise, based on the spread of supervisor experience across the score ranges (Table 2). We did not ask about our educators’ professional development training. Although potentially interesting, this information was tangential to our primary goal of assessing current workplace practice against recommended behaviours. Given that education paradigms have changed considerably across time, and that educator behaviour may partly reflect methods used when they were learners, the observed variability in feedback approaches highlights the need for continuing professional development that focuses on recent advances. The lack of striking differences in scores between professions suggests that feedback skills within formal encounters may be more similar than different. Hence feedback literacy training could, at least in part, be designed for educators across the health professions, allowing significant efficiencies. Nevertheless, the extent to which these skills vary within informal feedback encounters and across different contexts requires more study. Practising clinicans are responsible for the majority of health professions training (both senior students and junior clinicians) and yet specified standards for their education and training role are rare. In contrast health professionals spend many years training and being carefully assessed on their clinical skills.

The aim of our research is to assist educators in generating high quality learner-centred feedback, by developing descriptions of educator behaviours that could engage, motivate and enable learners to improve. It may well be that once clinicians have the opportunity to consider the recommended behaviours, it would be relatively easy for them to introduce missing elements into their practice. One strategy that might be valuable for educators would be to video their feedback with a learner and subsequently use the list to systematically analyse their own behaviours. This would enable educators to also engage in reflective learning and goal setting [57, 58]. In addition, exemplars of supervisors’ phrases or videos re-enacting quality feedback practices may help educators to translate the principles of high quality feedback into new rituals. The set of behaviours is comprehensive however it could be useful to prioritise or summarise them, as 25 recommended behaviours may seem overwhelming, especially to new educators.

### Study strengths and limitations

Strengths of our study include self-recorded video-observations of authentic feedback episodes in routine clinical practice, to reveal ‘what actually happens’ and target the top level of Miller’s framework for competency assessment. Participants involved a diverse group of clinical educators, characteristic of hospital practice. The educators’ feedback practices were systematically analysed utilising an empirically derived, comprehensive set of 25 observable educator behaviours.

There are a number of limitations to our study. The small sample of 36 participants were from a single health service, although it is one of the largest in Australia with multiple hospitals. Participants volunteered (which may have resulted in a subset of educators and learners with stronger skills than those who did not volunteer) and participants recorded their own performances, potentially making our data overly optimistic. These factors limit the generalisability of our findings. In the application of the educator behaviour descriptions to the assessment of educator behaviour during feedback, there was some variation in rater consistency. One reason for this could be different interpretations of the educator behaviour descriptions. In future research, attention will be directed to refining the descriptions of observable behaviours and supporting information, accompanied by additional practice and discussion to optimise consensus amongst raters. Although video raters represented only two health professions (two physicians and four physiotherapists), which could raise the possibility that this might influence their analysis of educators’ behaviours beyond their own profession, we cannot see a plausible argument to support this. A number of educators used official feedback forms (from university, hospital or specialty college). Trying to complete these forms in accordance with their instructions, may have influenced educators’ conduct or may have distracted educators’ attention, as they can be quite cognitively demanding. However, there are no compelling reasons why best practice in feedback could not occur in parallel with any learner assessment rubric. In addition, educator-learner pairs could have had earlier feedback conversations, during which some of the quality feedback behaviours may have occurred, particularly relating to setting up expectations and establishing trust, but were not captured on video.

## Conclusions

Our study showed that during formal feedback sessions, educators routinely provided their analysis of the learner’s performance, described how the task should be performed, and were respectful and supportive within the conversation. These are all valuable and recommended components of quality feedback. Nevertheless, other desirable behaviours were rarely observed. Important elements that were often omitted included deliberately instigating a safe learning environment at the start of the feedback session (by explicitly articulating the purpose, expectations and likely structure of the session), encouraging self-assessment, activating the learner’s motivation and understanding, creating an action plan and planning a subsequent performance review. This suggests that many advances in feedback research, regarding the importance of assisting learners to understand, incorporate and act on performance information, have not impacted routine clinical education. Our research clarifies valuable targets for educator feedback skill development across the health professions education community. However further research is required to investigate whether implementing these recommended educator behaviours results in enhanced learner outcomes, as designed.

## References

[1] Carraccio C, Englander R, Van Melle E, Ten Cate O, Lockyer J, Chan MK (2016). Advancing competency-based medical education: a charter for clinician-educators. Acad Med.

[2] Miller GE (1990). The assessment of clinical skills/competence/performance. Acad Med.

[3] van der Vleuten CP, Schuwirth LW, Driessen EW, Dijkstra J, Tigelaar D, Baartman LK (2012). A model for programmatic assessment fit for purpose. Med Teach.

[4] Hattie J, Timperley H (2007). The power of feedback. Rev Educ Res.

[5] Kluger AN, DeNisi A (1996). The effects of feedback interventions on performance: a historical review, a meta-analysis, and a preliminary feedback intervention theory. Psychol Bull.

[6] Ericsson KA (2015). Acquisition and maintenance of medical expertise: a perspective from the expert-performance approach with deliberate practice. Acad Med.

[7] Ivers N, Jamtvedt G, Flottorp S, Young JM, Odgaard-Jensen J, French SD, et al. Audit and feedback: effects on professional practice and healthcare outcomes. Cochrane Database Syst Rev. 2012;(6):CD000259. 10.1002/14651858.CD000259.pub3 PMC11338587 22696318

[8] Veloski J, Boex JR, Grasberger MJ, Evans A, Wolfson DB (2006). Systematic review of the literature on assessment, feedback and physicians’ clinical performance: BEME guide no. 7. Medical Teacher..

[9] Molloy E, Boud D, Boud DME (2013). Changing conceptions of feedback. Feedback in higher and professional education.

[10] Carless D, Boud D, Molloy E (2013). Trust and its role in facilitating dialogic feedback. Feedback in higher and professional education.

[11] Blatt B, Confessore S, Kallenberg G, Greenberg L (2008). Verbal interaction analysis: viewing feedback through a different lens. Teaching and Learning in Medicine.

[12] Bardella IJ, Janosky J, Elnicki DM, Ploof D, Kolarik R (2005). Observed versus reported precepting skills: teaching behaviours in a community ambulatory clerkship. Med Educ.

[13] Ende J, Pomerantz A, Erickson F (1995). Preceptors' strategies for correcting residents in an ambulatory care medicine setting: a qualitative analysis. Acad Med.

[14] Frye AW, Hollingsworth MA, Wymer A, Hinds MA (1996). Dimensions of feedback in clinical teaching: a descriptive study. Acad Med.

[15] Hekelman FP, Vanek E, Kelly K, Alemagno S (1993). Characteristics of family physicians’ clinical teaching behaviors in the ambulatory setting: a descriptive study. Teaching and Learning in Medicine..

[16] Huang WY, Dains JE, Monteiro FM, Rogers JC (2004). Observations on the teaching and learning occurring in offices of community-based family and community medicine clerkship preceptors. Fam Med.

[17] Kogan JR, Conforti LN, Bernabeo EC, Durning SJ, Hauer KE, Holmboe ES (2012). Faculty staff perceptions of feedback to residents after direct observation of clinical skills. Med Educ.

[18] Urquhart LM, Ker JS, Rees CE (2018). Exploring the influence of context on feedback at medical school: a video-ethnography study. Adv Health Sci Educ Theory Pract.

[19] Molloy E, Delany C, Molloy E (2009). Time to pause: feedback in clinical education. Clinical education in the health professions.

[20] Johnson CE, Keating JL, Boud DJ, Dalton M, Kiegaldie D, Hay M (2016). Identifying educator behaviours for high quality verbal feedback in health professions education: literature review and expert refinement. BMC Medical Education.

[21] Sadler DR (1989). Formative assessment and the design of instructional systems. Instr Sci.

[22] Locke EA, Latham GP (2002). Building a practically useful theory of goal setting and task motivation. A 35-year odyssey. The American psychologist.

[23] Deci EL, Ryan RM (2000). The ‘what’ and ‘why’ of goal pursuits: human needs and the self-determination of behavior. Psychol Inq.

[24] Ten Cate TJ, Kusurkar RA, Williams GC (2011). How self-determination theory can assist our understanding of the teaching and learning processes in medical education. AMEE guide no. 59. Medical Teacher..

[25] Ten Cate OT (2013). Why receiving feedback collides with self determination. Adv Health Sci Educ.

[26] Wadsworth BJ (1996). Piaget’s theory of cognitive and affective development: foundations of constructivism.

[27] Kaufman DM, Cantillon P, Wood D (2010). Applying educational theory in practice. ABC of learning and teaching in medicine.

[28] Butler DL, Winne PH (1995). Feedback and self-regulated learning: a theoretical synthesis. Rev Educ Res.

[29] Nicol DJ, Macfarlane-Dick D (2006). Formative assessment and self-regulated learning: a model and seven principles of good feedback practice. Stud High Educ.

[30] Ramani S, Konings KD, Ginsburg S, van der Vleuten CPM. Twelve tips to promote a feedback culture with a growth mind-set: swinging the feedback pendulum from recipes to relationships. Med Teach. 2018:1–7. https://www.tandfonline.com/doi/full/10.1080/0142159X.2018.1432850. 10.1080/0142159X.2018.1432850 29411668

[31] Sargeant J, Lockyer J, Mann K, Holmboe E, Silver I, Armson H (2015). Facilitated reflective performance feedback: developing an evidence- and theory-based model that builds relationship, explores reactions and content, and coaches for performance change (R2C2). Acad Med.

[32] Sargeant J, Lockyer JM, Mann K, Armson H, Warren A, Zetkulic M (2018). The R2C2 model in residency education: how does it Foster coaching and promote feedback use?. Acad Med.

[33] Ende J (1983). Feedback in clinical medical education. J Am Med Assoc.

[34] Telio S, Ajjawi R, Regehr G (2015). The "educational alliance" as a framework for reconceptualizing feedback in medical education. Acad Med.

[35] Norman G (2010). Likert scales, levels of measurement and the “laws” of statistics. Adv Health Sci Educ.

[36] Molloy E, Van de Ridder M, Delany C, Molloy E (2018). Reworking feedback to to build better work. Learning and teaching in clinical contexts.

[37] Bindal T, Wall D, Goodyear HM (2011). Trainee doctors' views on workplace-based assessments: are they just a tick box exercise?. Medical Teacher..

[38] Fernando N, Cleland J, McKenzie H, Cassar K (2008). Identifying the factors that determine feedback given to undergraduate medical students following formative mini-CEX assessments. Med Educ.

[39] Holmboe ES, Yepes M, Williams F, Huot SJ (2004). Feedback and the mini clinical evaluation exercise. J Gen Intern Med.

[40] Pelgrim EAM, Kramer AWM, Mokkink HGA, Vleuten CPM (2012). Quality of written narrative feedback and reflection in a modified mini-clinical evaluation exercise: an observational study. BMC Medical Education..

[41] Rudolph JW, Simon R, Raemer DB, Eppich WJ (2008). Debriefing as formative assessment: closing performance gaps in medical education. Acad Emerg Med.

[42] Silverman J, Kurtz S (1997). The Calgary-Cambridge approach to communication skills teaching ii: the set-go method of descriptive feedback. Educ Gen Pract.

[43] Sargeant J, Mann K, Ferrier S (2005). Exploring family physicians' reactions to multisource feedback: perceptions of credibility and usefulness. Med Educ.

[44] Johnson CE, Molloy EK, Boud D, Ajjawi R, Dawson P, Tai J (2018). Building evaluative judgement through the process of feedback. Developing evaluative judgement in higher education assessment for knowing and producing quality work.

[45] Carless D, Boud D (2018). The development of student feedback literacy: enabling uptake of feedback. Assess Eval High Educ.

[46] Sargeant J, Mann K, Sinclair D, Van der Vleuten C, Metsemakers J (2008). Understanding the influence of emotions and reflection upon multi-source feedback acceptance and use. Adv Health Sci Educ.

[47] Bing-You RG, Paterson J, Levine MA (1997). Feedback falling on deaf ears: residents' receptivity to feedback tempered by sender credibility. Medical Teacher..

[48] Grenny J, Patterson K, Maxfield D, McMillan R, Switzler A (2013). Infuencer the science of leading change.

[49] Sargeant J, Mann K, Sinclair D, van der Vleuten C, Metsemakers J (2007). Challenges in multisource feedback: intended and unintended outcomes. Med Educ.

[50] Telio S, Regehr G, Ajjawi R (2016). Feedback and the educational alliance: examining credibility judgements and their consequences. Med Educ.

[51] Patterson K, Grenny J, McMillan R, Switzler A (2012). Crucial conversations: tools for talking when the stakes are high.

[52] Kaufman DM, Mann KV, Swanwick T (2014). Teaching and learning in medical education: How theory can inform practice. Understanding medical education evidence, theory and practice.

[53] Shute VJ (2008). Focus on formative feedback. Rev Educ Res.

[54] Voyer S, Cuncic C, Butler DL, MacNeil K, Watling C, Hatala R (2016). Investigating conditions for meaningful feedback in the context of an evidence-based feedback programme. Med Educ.

[55] LaDonna KA, Hatala R, Lingard L, Voyer S, Watling C (2017). Staging a performance: learners’ perceptions about direct observation during residency. Med Educ.

[56] Flinn JT, Miller A, Pyatka N, Brewer J, Schneider T, Cao CG (2016). The effect of stress on learning in surgical skill acquisition. Medical Teacher.

[57] Irby DM (2014). Excellence in clinical teaching: knowledge transformation and development required. Med Educ.

[58] Knight J (2014). Focus on teaching: using video for high-impact instruction.

